# Factors affecting household disaster preparedness in South Korea

**DOI:** 10.1371/journal.pone.0275540

**Published:** 2022-10-04

**Authors:** Yujeong Kim, Mi Young Kim

**Affiliations:** 1 College of Nursing, Research Institute of Nursing Science, Kyungpook National University, Daegu, Republic of Korea; 2 College of Nursing, Hanyang University, Seoul, Republic of Korea; University Hospital Heidelberg, GERMANY

## Abstract

This study examines current household disaster preparedness and identifies its predictors in South Korea. A structured online survey was administered to 1,243 participants quota-sampled by age and population from each administrative district. Based on the socio-ecological model, interpersonal factors (general characteristics, prior disaster experience, anxiety, dispositional optimism, perceived disaster risk, and disaster preparedness knowledge), institutional factor (front-line preparedness), community factor (community resilience), public policy factor (governmental preparedness), and household disaster preparedness were measured. The data were analyzed using descriptive statistics, Mann–Whitney U test, Kruskal–Wallis test, Bonferroni test, Pearson’s correlation coefficients, and multiple regression. The predictors of household disaster preparedness were occupation, economic status, prior disaster experience, anxiety, disaster preparedness knowledge, front-line preparedness, and community resilience. The most potent predictor of household disaster preparedness was community resilience. Our finding that community resilience, a community factor, has a greater impact on household disaster preparedness than personal factors calls for programs that promote such resilience. Further, continuous public education and campaigns are needed to increase public awareness of household disaster preparedness and to improve the public’s competency to prepare for potential disasters. This study raises the need for community programs for residents to increase household disaster preparedness knowledge and improve their competencies related to disaster response. This study is significant in highlighting the importance of community factors in improving household disaster preparedness amid the need to prepare for various types of disasters.

## Introduction

Disasters are defined as a serious disruption of the functioning of a community or a society causing widespread human, material, economic or environmental losses and impacts which exceeds the ability of the affected community or society to cope using its own resources [1]. World Health Organization reported that about 190 million people are affected by disasters each year [2]. In South Korea, 25 disasters occurred in 2020, which led to 1,091 casualties, including 998 deaths; the economic loss due to these disasters totaled 334.27 billion KRW [3]. Disasters cannot be overcome without external assistance, and they bring about substantial damage that cannot be redressed using existing resources [4].

The growing severity of damages caused by disasters worldwide has accentuated the importance of disaster risk management, which calls for governmental intervention [5]. Existing policies have focused on enhancing disaster management competencies of organizations, such as increasing local governments’ disaster management competence, improving disaster management-related systems, enhancing disaster management planning abilities, and highlighting the importance of local governments in the disaster management process and improving their roles and functions [6]. Various disasters have brought about changes in disaster policies in South Korea as well, and there has been a demand for efforts to strengthen disaster response competency [7].

As disaster risk—in all of its dimensions of vulnerability, capacity, exposure of persons and assets, hazard characteristics, and the environment—is emphasized in disaster risk management [8], disaster preparation and management require not only national endeavors but also individuals’ voluntary involvement for disaster preparedness [9, 10]. Experts stressed that individuals would require partial or complete self-sufficiency for at least 72 hours following a disaster [10]. Each household must be equipped with an emergency kit and emergency food stock. Family emergency plans that outline how to contact and meet other family members during a crisis should be established. In contrast to the heated interest in the importance of governmental measures for disaster management, there is low awareness of household disaster preparedness in South Korea [9]. Thus, it is important to examine the current level of household disaster preparedness in South Korea and identify its predictors.

Some studies have attempted to identify the predictors of household disaster preparedness. A literature review [11] found that demographic characteristics, prior disaster experience, psychosocial factors, and disaster preparedness knowledge are the major predictors of personal disaster preparedness. Disaster management involves interpersonal, community, and government-related factors, and these factors are all mutually linked [12]. As suggested by the growing worldwide effort to connect families, schools, and communities to prepare for disasters [4, 13], community factors must be taken into consideration in interpersonal and governmental disaster preparedness. Considering these predictors of disaster preparedness, we applied the social-ecological model (SEM) [14], which posits that behavior is shaped by interactions at and impacts from different levels. Regarding the process of setting factors that affect disaster preparedness, we selected factors based on the SEM model.

Therefore, this study identifies the interpersonal, institutional, community, and public policy factors that affect household disaster preparedness based on SEM. More specifically, interpersonal factors comprise demographic factors, prior disaster experience, anxiety, dispositional optimism, perceived disaster risk, and disaster preparedness knowledge. Institutional factor refers to front-line preparedness, and community factor includes community resilience. Finally, public social factor includes governmental preparedness. Through this study, we ultimately aim to present foundational data for developing intervention programs, education programs, campaigns, and policies that enhance household disaster preparedness.

## Materials and methods

### Design

A cross-sectional design was used to examine household disaster preparedness and its predictors in South Korea.

### Participants

The participants were quota-sampled by age and population from each administrative district at a 1:1 sex ratio and recruited by a professional research company. In this study, the age of adult participants was allocated in proportion to the population. Thus, based on the standard population calculation criteria of the Korean government’s national health statistics [15], the participants of this study were aged between 19 and 69 years and resided in South Korea. Data were collected from July 13, 2021, to July 19, 2021, using an online survey. The participants who accessed the survey link were permitted to proceed with the survey only when they read the information and online consent form regarding this study and provided consent to participate. Only participants who voluntarily indicated their written consent to participate by checking “I agree” were allowed to proceed with the online questionnaire. The survey took about 10–15 minutes to complete. Sample size was determined using the G*Power 3.1 software. With an effect size of .02, power of .80, an α of .05, and a maximum of 18 predictors, the minimum sample size was calculated to be 1,022. Considering potential withdrawals, the survey link was sent to 2,208 individuals. A total of 1,243 people completed the survey.

The study was approved by the Kyungpook National University’s Institutional Review Board (no. KNU-2021-0106). Ethical issues regarding plagiarism, informed consent, misconduct, data fabrication and/or falsification, double publication and/or submission, and redundancy have been observed by the authors.

### Instruments

The survey was measured through the database of a professional research company in charge of the online survey. Information regarding the general characteristics of the participants were collected, including gender, age (19–69 years), education level, marital status, number of children, occupation, economic status, subjective health status, and prior disaster experience. Personal factors included anxiety, dispositional optimism, perceived disaster risk, and disaster preparedness knowledge. Institutional factor included front-line preparedness, community factor included community resilience, and public policy factor included governmental preparedness.

Anxiety was assessed using the Korean version of the Generalized Anxiety Disorder-7 [16]. The scale comprises seven items related to worrying too much, having bad feelings about what is about to happen, becoming irritable, losing concentration, and being restless. Each item is rated on a four-point Likert scale from 0 “Not at all” to 3 “Nearly every day.” A higher score indicates a higher level of anxiety. The Cronbach’s α was .92 at the time of development and .91 in this study.

Dispositional optimism was measured using the Life Orientation Test-Revised (LOT-R) [17]. The 10-item LOT-R is rated on a five-point Likert scale, and four items are filter items that are not scored. Negatively worded items are reverse-scored, and the total score ranges from 6–30. A higher score indicates higher dispositional optimism. The Cronbach’s α was .78 at the time of development and .73 in this study.

Perceived disaster risk refers to the degree of the perceived possibility of a disaster and related risk and was assessed using the instrument developed by Rosenstock et al. [18]. Each of the eight items is rated on a five-point Likert scale from 1 “Strongly disagree” to 5 “Strongly agree.” A higher score indicates higher perceived disaster risk. The Cronbach’s α was .87 at the time of development and .85 in this study.

Disaster preparedness knowledge was measured using one item: “Do you know what to do in case of a disaster?” Each item is rated on a four-point Likert scale from 1 “Don’t know about it at all” to 4 “Know very much about it.” A higher score indicates higher disaster preparedness knowledge.

Front-line preparedness was measured using the instrument developed by Lee and Lemyre [19]. The original tool assesses the level of disaster preparedness of first responders (e.g., police officers, emergency medical technicians, firefighters), hospital and health care services, non-governmental organizations (e.g., Red Cross, Salvation Army), and local community organizations (e.g., religious organizations, community clubs) using one item for each group. Each of the four items is rated on a five-point Likert scale from 1 “Strongly disagree” to 5 “Strongly agree.” A higher score indicates higher front-line disaster preparedness. The Cronbach’s α was .77 at the time of development and .74 in this study.

Community resilience refers to the community’s ability to reduce risk, suppress the impact of a disaster, minimize confusion across the society, and engage in restoration activities to alleviate the impact of potential disasters in the future [20]. Community resilience was measured using the Conjoint Community Resiliency Assessment developed by Leykin et al. [21] and adapted by the National Disaster Management Research Institute [22]. Each of the 10 items is rated on a five-point Likert scale from 1 “Strongly disagree” to 5 “Strongly agree.” The Cronbach’s α was .85 at the time of development and .93 in this study.

Governmental preparedness was measured using the instrument developed by Lee and Lemyre [19]. The original tool measures terror response by the federal government, provincial government, and municipal government using one item for each group. In this study, we measured the disaster preparedness of the government, province, and city in reflection of the administrative districts of South Korea. Each item is rated on a five-point Likert scale from 1 “Strongly disagree” to 5 “Strongly agree.” A higher score indicates higher perceived governmental preparedness. The Cronbach’s α was .81 at the time of development and .88 in this study.

Household disaster preparedness was measured using the Emergency Preparedness Checklist developed by the Federal Emergency Management Agency [23] and modified by Blessman et al. [24]. This checklist comprises 21 items regarding family emergency contacts, emergency kits, and emergency food boxes. All items are yes (1) or no (0) questions. A higher summed score indicates greater household disaster preparedness. The Kuder-Richardson 20 was .82 in this study.

### Statistical analysis

The collected data were analyzed using the SPSS 22.0 software (IBM Corp., Armonk, NY, USA). The general characteristics of the participants were analyzed using descriptive statistics—frequency, percentage, mean, and standard deviation. The major study variables were presented as mean, standard deviation, maximum, and minimum. The differences in household disaster preparedness according to demographic characteristics were analyzed using the Mann–Whitney U test, Kruskal–Wallis test, and Bonferroni test. The relationships between household disaster preparedness and other study variables were analyzed with Pearson’s correlation. The predictors of household disaster preparedness were identified using stepwise multiple regression with variables that were significant in the univariate analyses.

## Results

Of the 1,243 participants, 50.8% were male and 49.2% were female. The mean age was 44.35 years. The highest education was college or higher for 75.9% of the participants. A total of 62.2% were married, and 56.1% had at least one child. The most common occupation was office worker (35.2%), followed by unemployed (including housewives and students; 30.3%). Most participants considered themselves to be of moderate economic status (64.6%), and 70.6% perceived themselves to be in moderate health. The majority (85.4%) did not have prior disaster experience (Table 1).

**Table 1:** Differences in household disaster preparedness according to the general characteristics of the participants (*N* = 1,243).

Variables	Categories	n (%)	Household disaster preparedness	
		M±SD	M±SD	*t* or *F*(*p*)
				Bonferroni
Gender	Male	631 (50.8%)	5.45±3.96	0.16 (.685)
	Female	612 (49.2%)	5.25±3.61	
Age (years)	19–29^a^	237 (19.1%)	5.11±4.08	20.37 (< .001)
	30–39^a^	220 (17.7%)	5.29±4.45	
	40–49^a^	275 (22.1%)	5.01±3.69	
	50–59^a,b^	282 (22.7%)	5.59±3.35	
	60–69^b^	229 (18.4%)	5.79±3.38	a<b
	Total	44.35±13.29	5.35±3.79	
Education level	High school or less	229 (24.1%)	5.54±3.77	0.99 (.324)
	College or higher	944 (75.9%)	5.29±3.80	
Marital status	Unmarried	470 (37.8%)	4.99±4.22	23.20 (< .001)
	Married	773 (62.2%)	5.58±3.50	
Children	No	546 (43.9%)	5.01±4.11	22.69 (< .001)
	Yes	697 (56.1%)	5.62±3.50	
Occupation	Managers, experts^a^	178 (14.3%)	6.06±3.96	16.31 (.012)
	Office workers	438 (35.2%)	5.00±3.52	
	Sales and service workers^c^	122 (9.8%)	5.48±4.19	
	Technical workers and labor workers^d^	128 (10.3%)	5.70±4.02	a>b
	Unemployed (housewife, student, etc.)^e^	377 (30.3%)	5.27±3.77	
Economic status	Low^a^	394 (31.7%)	4.76±3.85	30.18 (< .001)
	Moderate^b^	803 (64.6%)	5.54±3.68	
	High^b^	46 (3.7%)	7.15±4.43	b>a
Subjective health status	Bad^a^	146 (11.7%)	4.99±3.82	7.50 (.024)
	Moderate^b^	877 (70.6%)	5.26±3.70	
	Good^c^	220 (17.7%)	5.95±4.09	b>a
Prior disaster experience	No	1,062 (85.4%)	5.25±3.77	8.29 (.004)
	Yes	181 (14.6%)	5.97±3.86	

Household disaster preparedness was found to be significantly higher among the participants aged 60–69 years than other age groups (*F* = 20.37, *p* < .001) and significantly higher among married people (*t* = -23.20, *p* < .001) than single people. Families with at least one child (*t* = 22.69, *p* < .001) scored significantly higher compared to those with none. By occupation, managers and experts showed significantly higher household disaster preparedness than office workers (*F* = 16.31, *p* = .012). Further, household disaster preparedness was significantly high among those of high economic status (*F* = 30.18, *p* < .001) and good health status (*F* = 7.50, *p* = .024). People with prior disaster experience showed significantly higher household disaster preparedness (*t* = 8.29, *p* = .004). Household disaster preparedness did not significantly vary according to gender or education level (Table 1).

The mean interpersonal factor scores were 4.59 for anxiety, 19.93 for dispositional optimism, 3.98 for perceived disaster risk, and 2.73 for disaster preparedness knowledge. The mean front-line preparedness (institutional factor) score was 3.09. The mean community resilience (community factor) score was 2.92. The mean governmental preparedness (public policy factor) score was 2.95, and the mean household disaster preparedness score was 5.35. Most participants (59.9%) were “unprepared” for a disaster (score ≤ 5), followed by “minimally prepared” (31.5%), “well prepared” (6.4%), and “maximally prepared” (2.2%) (Table 2).

**Table 2:** Level of study variables (*N* = 1,243).

Level	Variables	M±SD, n (%)	Range	Min	Max
Interpersonal factor	Anxiety	4.59±4.31	0–21	0.0	21.0
	Dispositional optimism	19.93±3.37	6–30	6.0	30.0
	Perceived disaster risk	3.98±0.51	1–5	1.0	5.0
	Disaster preparedness knowledge	2.73±0.52	1–4	1.0	4.0
Institutional factor	Front-line preparedness	3.09±0.65	1–5	1.0	5.0
Community factors	Community resilience	2.92±0.64	1–5	1.0	5.0
Public policy factor	Governmental preparedness	2.95±0.83	1–5	1.0	5.0
Household disaster preparedness		5.35±3.79	0–21	0.0	21.0
	Unprepared (0–5)	744 (59.9%)	-		
	Minimally prepared (6–10)	392 (31.5%)	-		
	Well prepared (11–15)	79 (6.4%)	-		
	Maximally prepared (16–21)	28 (2.2%)	-		

The predictors of household disaster preparedness were analyzed with stepwise multiple regression (Table 3). In model 1, demographic characteristics and interpersonal factors that significantly varied according to household disaster preparedness were included as the independent variables. In model 2, the institutional factor (front-line preparedness), community factor (community resilience), and public policy factor (governmental preparedness) were added. Before conducting a regression analysis, autocorrelation and multicollinearity of independent variables were reviewed. The Durbin-Watson statistic was close to 2, at 2.03 and 2.00 in models 1 and 2, respectively, confirming the absence of autocorrelation. Tolerance was above 0.1 in both models 1 and 2. Multicollinearity among independent variables was tested using the variance inflation factor (VIF), and a VIF of below 10 (1.01–4.05 for model 1, 1.02–4.07 for model 2) confirmed the absence of multicollinearity.

**Table 3:** Factors influencing household disaster preparedness (*N* = 1,243).

Variables	Model 1				Model 2			
	B	SE	β	t (*p*)	B	SE	β	t (*p*)
(constant)	-2.18	1.05		-2.07 (.038)	-4.37	1.21		-3.61 (< .001)
Occupation (ref = office worker)								
Managers, experts	0.69	.33	.06	2.13 (.034)	0.63	.32	.06	1.98 (.048)
Technical workers and labor workers	0.79	.37	.06	2.13 (.033)	0.58	.36	.05	1.63 (.104)
Economic status (ref = low)								
Moderate	0.59	.25	.07	2.41 (.016)	0.53	.24	.07	2.26 (.024)
High	2.10	.60	.11	3.62 (< .001)	2.16	.59	.11	3.84 (< .001)
Prior disaster experience (ref = no)	0.62	.30	.06	2.10 (.036)	0.71	.29	.07	2.47 (.014)
Anxiety	0.09	.03	.11	3.40 (.001)	0.09	.03	.10	3.40 (.001)
Disaster preparedness knowledge	1.61	.20	.22	7.88 (< .001)	1.33	.20	.18	6.66 (< .001)
Front-line preparedness					0.56	.23	.10	2.46 (.014)
Community resilience					1.26	.22	.21	5.68 (< .001)
	R^2^ = .09, Adjusted R^2^ = .08				R^2^ = .15, Adjusted R^2^ = .14			
	*F* (*p*) = 8.39 (< .001)				*F* (*p*) = 12.23 (< .001)			

In model 1, occupation, economic status, prior disaster experience, anxiety, and disaster preparedness knowledge were the significant predictors of household disaster preparedness. In other words, managers and experts, technical workers, and labor workers demonstrated significantly higher household disaster preparedness than office workers. Further, people of moderate or high economic status demonstrated significantly higher household disaster preparedness than those of low economic status, and people with prior disaster experience showed significantly higher household disaster preparedness than those without such experience. Additionally, individuals with high anxiety and greater disaster preparedness knowledge showed significantly higher household disaster preparedness.

In model 2, occupation (managers and experts), economic status (moderate, high), prior disaster experience, anxiety, disaster preparedness knowledge, front-line preparedness, and community resilience were identified as significant predictors of household disaster preparedness. Of these, community resilience (β = .21, *p* < .001) was the most powerful predictor, followed by disaster preparedness knowledge (β = .18, *p* < .001).

## Discussion

This study examined the current levels of household disaster preparedness in South Korea as well as its predictors. Our results showed that 59.9% of the participants were “unprepared,” and 31.5% were “minimally prepared” for a disaster. This result is similar to a study in which most residents near a chemical weapons storage depot had neither a family disaster plan nor supplies [25]. However, the rates in South Korea are low compared to the rate of self-assessed preparedness of 59% in 2019 and 44% in 2021 [26]. The rate of preparedness in South Korea is similarly low as the results of a report stating that 75% of US public health employees were “minimally prepared” or “not prepared” for a disaster [24]. However, as the data in this study were collected on an individual basis, we suggest that future studies should analyze data collected on a household-based cluster.

Predictors of household disaster preparedness were analyzed in terms of interpersonal factors, institutional factors, community factors, and public policy factors based on SEM. First, among interpersonal factors, occupation, economic status, prior disaster experience, anxiety, and disaster preparedness knowledge were the significant predictors of household disaster preparedness. In terms of occupation, managers and experts had higher household disaster preparedness than office workers. Those of moderate or high economic status showed higher household disaster preparedness than those of low economic status. The results show that people of higher socioeconomic status are more prepared for disasters. This is in line with previous findings showing that people of high socioeconomic status (high income, own a house) were more prepared for disasters by storing food, water, or first-aid supplies at home [25, 27]. Moreover, this result is consistent with a study on US and international samples, in which household disaster preparedness increased with rising income [28–30]. Thus, low-cost preparation measures need to be developed such that socioeconomically disadvantaged households can also prepare for disasters. Preparing for potential events in the future requires time and money. Therefore, it is necessary to propose time-efficient and low-cost means to prepare for disasters.

Prior disaster experience was identified as an interpersonal factor influencing household disaster preparedness, consistent with previous findings that prior disaster experience is associated with household disaster preparedness [30–32]. However, some studies proposed that the association between prior disaster experience and household disaster preparedness is unclear [11]. It can be inferred that prior disaster experience does not simply promote household disaster preparedness but instead serves as a motivator that drives individuals to prepare for disasters. Accordingly, interventions that motivate individuals to prepare for potential disasters in the future are essential during the process of restoration and recovery from a disaster.

Anxiety, an interpersonal factor, was also a significant predictor of household disaster preparedness. If anxiety is viewed in the spectrum of psychological stress, this result supports previous findings that psychological stress affects household disaster preparedness planning [33]. However, it cannot be definitively concluded that psychological stress influences household disaster preparedness [34] which suggests that psychological stress (including anxiety) itself does not directly lead to adaptive behaviors such as household disaster preparedness but, rather, that individuals’ actions may vary depending on how the anxiety is processed and supported. Hence, interventions that facilitate the translation of anxiety to positive adaptive behaviors, such as disaster preparedness, are required.

Disaster preparedness knowledge, also an interpersonal factor, was a significant predictor of household disaster preparedness. This is consistent with a previous report that 75% of individuals mentioned “lack of knowledge” as a major barrier to disaster preparedness [35]. However, in another study, the participants mentioned that they do not specifically know how to prepare for disasters even if they have knowledge [36]. In other words, while lacking relevant knowledge would hinder preparing for disasters, knowledge alone does not necessarily help individuals create their emergency kits or establish emergency plans. Thus, along with increasing the public’s overall knowledge regarding disaster preparedness, specific action plans and easy guidelines that help individuals prepare for disasters based on their knowledge need to be developed and implemented.

Front-line preparedness, an institutional factor, significantly predicted household disaster preparedness in this study. This suggests that individuals and societies interact during the process of disaster preparedness and that this ultimately influences household disaster preparedness. Disasters are managed in the process of prevention, preparation, response, and restoration, and this process of management involves an interaction among the government, local residents, and unofficial actors [37]. Community residents are both the targets of disaster management and agents who play decisive roles in the success of disaster management by the government as they interact with disaster management-related administration and policies during dynamic disaster management governance. Therefore, household disaster preparedness will be greatly hampered if an individual perceives the governmental, organizational, and community preparation and response measures as ineffectual. The Federal Emergency Management Agency [26] examined disaster awareness, experience, risk perception, and efficacy as potential factors associated with preparedness and reported that household disaster preparedness declined with decreasing risk perception and efficacy. According to the Federal Emergency Management Agency [26], most respondents in the previous study experienced the impact of disasters but lacked conviction that they were capable of preparing for disasters and that their arrangements would help them be equipped for disasters. In other words, individuals must believe that their preparations for disasters will be practically useful and effective, and to this end, communities must be able to instill trust in their residents with effective front-line preparedness.

In terms of community factors, community resilience was identified as the most potent predictor of household disaster preparedness. The results were consistent with those of a study conducted in northwest China, in which disaster preparedness was associated with perceived community resilience [38]. The United Nations Office for Disaster Risk Reduction [39] launched the Making Cities Resilient 2030 campaign emphasizing the importance of providing support, sharing knowledge and experience, establishing a learning network among cities, introducing technological expertise, connecting various dimensions of the government, and building partnerships to make cities resilient. As governmental intervention and capacity are highlighted due to the complex and uncertain nature of disasters [5], individuals’ preparation alone cannot ensure proper preparedness and coping with disasters. Communities must instill a belief in their residents that their preparations for disasters will be effective [26], and strengthening the resilience of communities also impacts household disaster preparedness. Therefore, programs that contribute to community resilience are required. In the United States, for example, a recent program at the National Institute of Standards and Technology (NIST) is developing science-based methods, tools, and guidelines for community resilience [40]. The NIST Community Resilience Planning Guide provides a practical and flexible step approach to help community resilience by setting priorities and allocating resources to manage risks for their prevailing hazards [41]. Therefore, countries and communities need to share successful community resilience programs and strategies to strengthen community capacity for disasters.

In our study, governmental preparedness, a public policy factor, did not significantly predict household disaster preparedness. This suggests that household disaster preparedness is more heavily influenced by the individuals’ communities than by the central government. While the nation plays the primary role in reducing disaster risk, the responsibility and effort must be shared by local governments, the private sector, and other stakeholders. The main agents of disaster response have shifted from the central government to local governments and private sectors [42]; thus, it is essential to strengthen communities’ disaster preparedness competencies. Additionally, enhancing disaster management competencies at the organizational level is crucial, such as by strengthening the disaster management competencies of local governments, ameliorating relevant systems, improving disaster management planning, and highlighting the importance and enhancing the roles and functions of local governments in disaster management. SEM posits that a holistic and comprehensive multi-stage approach leads to better behavioral changes than individual-level psychosocial interventions [14]. Our results show that institutional and community factors are equally as important as interpersonal factors in promoting household disaster preparedness.

The limitations and future implications of this study are as follows. In this study, both four- and five-point Likert scales were used; hence, this may have caused a potential bias. Therefore, caution is required while interpreting the results of this study. As a household cluster survey was not conducted in this study, future studies should conduct household cluster data collection and multi-level model analyses.

## Conclusion

Our study showed that the majority of households in South Korea are not prepared for disasters. Institutional factors and community factors were as important as interpersonal factors in promoting household disaster preparedness. Community resilience (a community factor) was the most potent predictor of household disaster preparedness (β = .21, p < .001), followed by disaster preparedness knowledge (an interpersonal factor). Thus, there is a pressing need to increase household disaster preparedness. Doing so requires the implementation of community programs for residents to enhance community resilience. Furthermore, continuous education and campaigns are needed to increase the public’s disaster preparedness knowledge and improve their competencies for disaster response.

## Supporting information

S1 Appendix Data set. (XLSX)
